# Validity of self-reported use of sulphadoxine-pyrimethamine intermittent presumptive treatment during pregnancy (IPTp): a cross-sectional study

**DOI:** 10.1186/1475-2875-11-310

**Published:** 2012-09-05

**Authors:** Fatuma Namusoke, Muhammad Ntale, Mats Wahlgren, Fred Kironde, Florence Mirembe

**Affiliations:** 1 Department of Obstetrics and Gynaecology, Makerere University, Kampala, Uganda; 2 Department of Microbiology, Tumor and Cell Biology (MTC), Karolinska Institute, Stockholm, Sweden; 3 Department of Biochemistry, Makerere University, Kampala, Uganda; 4 Department of Chemistry, Makerere University, Kampala, Uganda

**Keywords:** Pregnancy malaria, Intermittent presumptive treatment, Self-reported data

## Abstract

**Background:**

Malaria in pregnancy is a major health problem that can cause maternal anaemia, stillbirth, spontaneous abortion, low birth weight and intra-uterine stunting. The WHO recommends use of sulphadoxine-pyrimethamine (SP) for intermittent preventive treatment of malaria during pregnancy (IPTp) in endemic areas. Towards monitoring and assessing IPTp coverage in the population, the Roll Back Malaria Partnership recommends the use of self-reported data. The aim of this study was to assess the validity of self-reported IPTp by testing for sulphadoxine in maternal blood at delivery.

**Methods:**

Two hundred and four pregnant women were consented and enrolled in a cross-sectional study in Mulago National Referral Hospital in Kampala Uganda. - Participants who reported a history of taking sulpha-containing drugs like co-trimoxazole , those who were not sure of dates relating to last menstrual period or who took IPTp within the first 20 weeks of gestation were excluded from the study. Data on demographic characteristics, obstetric history, and delivery outcome were collected. At birth, maternal venous blood was taken off aseptically and used to make thick blood smears for malaria parasites and plasma for determining sulphadoxine using high performance liquid chromatography (HPLC).

**Results:**

Of 120 participants who self reported to have used IPTp, 35 (29.2%) tested positive for sulphadoxine by HPLC, while 63 (75%) of 84 patients who reported not having used IPTp tested negative for sulphadoxine. Participants possessing post-primary education were more likely to have reported using IPTp. The low agreement (kappa coefficient = 0.037) between self-report and actual presence of the drug in the blood casts doubt on the validity of self-reported data in estimating IPTp coverage.

**Conclusions:**

The results of this study question the accuracy of self-reported data in estimating IPTp coverage in the population. More studies on validity of self reported data are recommended. Since the validity of IPTp self reports is vital for guiding policy on malaria control in pregnancy, ways should be sought to improve accuracy of the information from such reports.

## Background

Malaria in pregnancy is a major health problem affecting both mother and unborn child [1]. Worldwide, malaria affects 300–500 million people and causes nearly one million deaths annually, mostly in children and pregnant women in sub-Saharan Africa [2]. Pregnancy malaria is associated with abortion, stillbirth, low birth weight, and intra-uterine foetal retardation [3]. The WHO Roll Back Malaria Partnership (RBM) recommends reducing the burden of pregnancy malaria by three established interventions: prompt management of malaria cases, intermittent preventive treatment of malaria in pregnancy (IPTp), and extensive use of insecticide-treated bed nets (ITNs) in endemic countries [4].

The use of IPTp regimen consists of giving two or three curative doses of SP (each dose comprising 1500 mg sulphadoxine plus 75 mg pyrimethamine) during pregnancy [5]. While, SP has a good safety profile in pregnancy, it is not normally administered in the first trimester and after 36 weeks of amenorrhoea or gestation because of fear of congenital abnormalities in early pregnancy and kernicterus later. In Uganda, according to the Uganda Demographic Health survey 2011 [6], the IPTp coverage is 67.5% while in Mulago Hospital, it was 41% in 2005 despite a high coverage (98%) in the antenatal care unit of the same hospital [7]. Sulphadoxine/pyrimethamine-resistant *Plasmodium falciparum* strains have been widely reported in Uganda and SP is now largely reserved for use as IPTp [8]. This is so because in semi-immune individuals, anti-malarial drugs with partial parasite resistance such as SP are still effective for intermittent presumptive treatment [9].

The Roll Back Malaria Partnership recommends using self reported data to determine IPTp coverage in a population. For this, self-reported data is collected from women who have had delivery of a baby in a year or more prior to the survey [10]. However, self-reported information on drug use has been found to be prone to bias and its validity questioned [11,12]. Doubts about the validity of self-reported drug use arise from several factors which adversely affect the accuracy of patients’ reporting, including selective recall, unawareness of the diagnosis or unwillingness to report [13]. Yet, accurate data on IPTp coverage is key to the design and implementation of effective control measures against the harmful effects of malaria to pregnant women and the newborn. The aim of this study was to assess the validity of self-reports on IPTp use by detecting sulphadoxine in maternal blood at the time of delivery.

## Methods

### Study site

The study was carried out in Mulago Hospital, which serves as Uganda’s National Referral Hospital and is located in the capital city of Kampala. Situated at 1,300–1,500 m above sea level close to the Equator, Kampala has a tropical climate with rainfalls throughout the year. There is stable *P. falciparum* transmission in 95% of Uganda. The remaining 5% of the country, mainly the highland areas with altitudes >1,600 m, experiences low and unstable malaria transmission. Kampala has low to intermediate malaria transmission with frequency peaks toward the end of the two major rain seasons (March to May and August to November). The national treatment guidelines recommend that pregnant women should receive at least two doses of SP to prevent malaria and its effects. At time of this study, HIV prevalence in the Ugandan population aged 15 to 49 years was 6.4% and prevalence among admitted patients at Mulago Hospital was 10%. Pregnant mothers with known HIV infection are expected to follow national guidelines of weekly trimethoprim-sulphamethoxazole (co-trimoxazole) prophylaxis to prevent opportunistic infections.

### Study population and data collection

Two hundred and four pregnant women admitted at Mulago National Referral Hospital labour suite were enrolled into a cross–sectional study after informed oral and written consent. Data on pregnancy history, socio-economic indicators and pregnancy outcomes was collected using a pre-coded standardized questionnaire. Key aspects recorded included area of residence, age, marital status, occupation, education, parity, visits to antenatal clinic (ANC) and bed net use. Birth weight of baby was determined after delivery. In addition, information on use of IPT for prevention of malaria during that pregnancy, the drug administered, number of SP doses taken, history of taking sulpha-containing drugs such as co-trimoxazole , history of fever during pregnancy, and use of anti-malarial drugs was recorded. The date on which the SP was taken was noted in the questionnaire. In cases where the patient was not able to state the dates with certainty, it was then recorded as the 15^th^ day of that particular month. This information was used to estimate the gestation age corresponding to when the SP was taken.

All ethical aspects of the study were approved by the Makerere University Faculty of Medicine Research and Ethics Committee and the Uganda National Council of Science and Technology (UNCST).

### Sample collection and laboratory analysis

Before delivery of baby, mother’s venous blood was collected for microscopy to detect parasites, for haemoglobin estimation and sulphadoxine (SDX) detection. Blood was collected in EDTA anticoagulant containing tubes, centrifuged, plasma separated and stored at −70°C until drug assays.

### Malaria parasite detection

Thick blood smears were made from the maternal venous blood and the cord blood. These were then stained with Giemsa and examined microscopically by two trained workers. In case of discrepancy, a third microscopist examined the smears.

### HPLC analysis

Plasma drug levels were assayed using the high performance liquid chromatography (HPLC) facility at the Department of Pharmacology and Therapeutics, College of Health Sciences, Makerere University, Kampala, Uganda. Sulphadoxine was used as a proxy for SP. The HPLC analysis (UV) was carried out according to the method described by Bergqvist *et al.*[14]. Sulphamethaxazole was used as the internal standard. The limit of quantification for SDX was 15 μM. Basing on average Cmax for SDX of 260 μM and assuming a half life (T_1/2_) for SDX of 6 to 9 days [15,16], it is calculated that SDX is detectable in blood from few hours after intake of SP until 7 to 9 weeks. Therefore, participants who reported as having taken IPTp before 20 weeks of their pregnancy were excluded from HPLC analysis. In addition, HIV-positive participants who reported being on co-trimoxazole prophylaxis were excluded since this antifolate combination is similar to SP and is a sulpha-containing drug that can interfere with HPLC detection of SDX. Further, as it was important to know the time interval between IPTp intake and blood sampling at delivery, the blood specimens of individuals who were unsure of the month of their last menstrual period (period of amenorrhea) were excluded. All plasma specimens were analysed twice along with calibration standards and quality controls. To prevent bias, the HPLC analysts were blinded to the data of self-reported IPTp uptake and composition of quality control samples.

### Data analysis

Data was cleaned, coded and entered into Microsoft Access 2007. Summary statistics, Chi-square tests, multivariate analysis and graphs of residual plasma concentrations of SDX were carried out using SPSS. Agreement or disagreement between self-report and HPLC results on actual detection of SDX in blood at delivery was determined by calculating kappa coefficients [17]. A kappa value of 0.1 to 0.40 was considered poor-to-fair agreement, a kappa value of 0.41 to 0.60 was considered moderate agreement, and a kappa value of 0.61 to 0.80 was considered substantial agreement, while a kappa value of 0.81 to 1.00 was considered excellent agreement.

## Results

**Figure 1:**
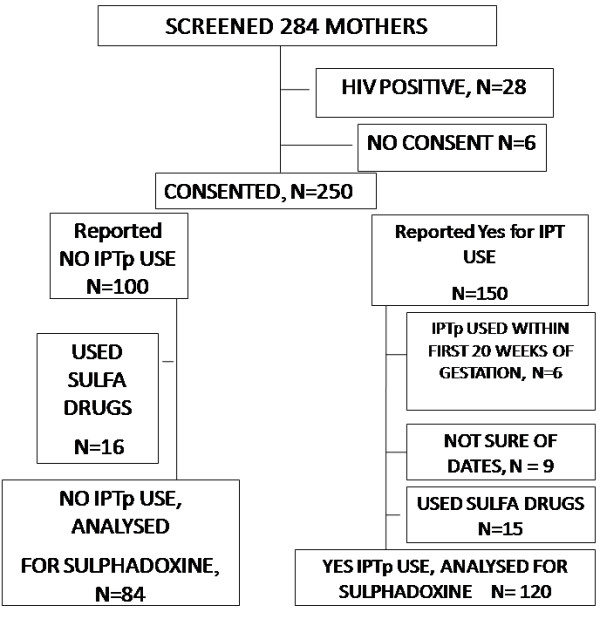
Participant flow chart.

In a study to assess the validity of self-reported data on the use of anti-malarial IPTp, 284 pregnant participants were screened between September 2008 and July 2009 (Figure 1).

**Figure 2:**
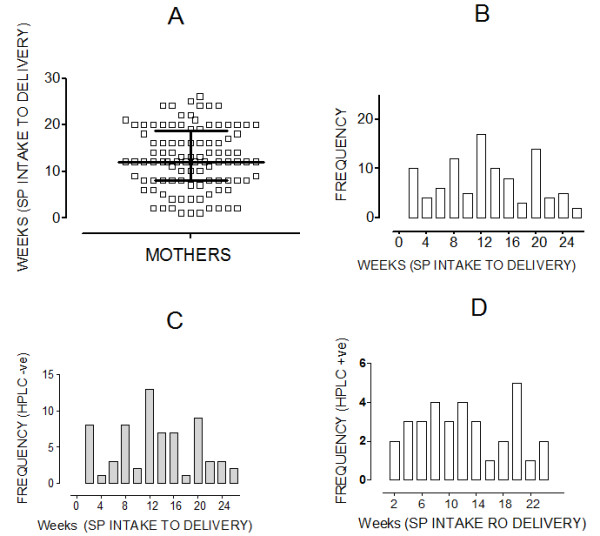
**Reported time interval between SP administration and delivery.** At baby delivery, women donated blood to measure sulphadoxine in their blood by HPLC (UV) and were interviewed to self-report on whether and when they received pyrimethamine-sulphadoxine (SP) during the pregnancy. Agreement between the HPLC result and self report was calculated by kappa analysis. The Figure shows: **A**. Variability in interval (weeks) between reported date of SP intake and baby delivery (median: 12 weeks, IQR: 8–18.8 weeks, see whiskers), **B**. Frequency distribution of the reported interval (SP input to delivery) for the mothers whose blood was analyzed for SDX. Histogram of reported interval (SP intake to delivery) for HPLC negative (**C**) and HPLC positive (**D**) mothers.

Majority (98.5%) of the participants attended antenatal clinic at least once during pregnancy as evidenced by self-report and presence of an antenatal clinic card. Approximately fifty nine percent of participants (n = 204) reported using IPTp during pregnancy, with 90% taking one dose of SP while 17.2% reported using an insecticide spray for controlling mosquito bites. From the self-reports on when the last SP dose closest to delivery was taken, the median reported interval between SP intake and baby delivery was computed as 12 weeks (IQR: 8–18.8); see Figure 2. Frequency distribution of the calculated interval between reported date of SP intake and baby delivery for the mothers who reported having used IPTp is shown in Figure 2 (histogram B). The frequency distribution of the same interval for mothers (n = 35) who were found to have detectable SDX in blood (Figure 2D) and those (n = 85) whose blood was negative for SDX (Fig 2C) are also shown. It can be seen that SDX was detected in blood of mothers whose self reports indicated SP intake before 9 weeks to baby delivery (Figure 2D) , a result suggesting that the reported dates of the IPT dose was incorrect since SDX would be undetectable by HPLC beyond two months after administration. On the other hand, the blood of more than 15 mothers who reported to have taken SP within 9 weeks preceding baby delivery lacked any detectable SDX (Figure 2C), likewise suggesting that the reported dates for when the SP doses were taken are inaccurate. Thus, the results suggest that the self reports were unreliable for finding out whether the patients used IPTp or not (Table 1) and for determining when the SP doses were taken (Figure 2). It is unlikely that HPLC assay errors are responsible for discrepancy with self-reports because SDX was detected both in blood of many patients reporting to have taken SP within two months before delivery and in specimens of others reporting the IPT dose intake to have occurred more than two months before delivery. If the self reports were accurate and the HPLC assay falsely detected SDX in blood specimens, then the vast majority of patients reporting use of IPT particularly within two months before delivery would have been SDX positive unlike what the results show (Table 1 and Figure 2C).

**Table 1:** Self-reported IPTp use and presence of sulphadoxine in blood

**Blood Sulphadoxine**	**Reported use of IPTp**		**Total**
	Yes	No	
Positive	35	21	**56 (m1)**
Negative	85	63	**148 (m0)**
Total	**120 (n1)**	**84 (n0)**	**204 (n)**

One hundred and sixty five (80.9%, n = 204) of the participants were resident in Kampala district, 18.1% were from nearby (approximately 10 to 20 km) Wakiso district, with the remainder from further away (approximately 20 to 40 km) in Mukono and Luwero districts. Most (64.2%) participants had some form of employment as businesswomen or self-employed individuals. Skilled workers, including professionals were 11.3% while 13.2% had no formal employment. The median age of the participants was 23 years (interquartile range: 20–27). The other details of the demographic characteristics of the study population are shown in Table 2.

**Table 2:** Demographic characteristics of study participants

***Variable***	***Frequency***	***(%) Percentage***
IPTp use		
Yes	120	58.8
No	84	41.2
ANC attendance		
Yes	201	98.5
No	3	1.5
IPTp doses taken		
1	108	90
2	10	8.3
3	2	1.7
Bed net use		
Always	165	80.9
Sometimes	17	8.3
Never	22	10.8
Bed net		
Insecticide-treated	72	35.3
Not treated	70	34.3
Don’t know	38	18.6
Folic acid use		
yes	150	73.5
no	54	26
Iron sulphate use		
Yes	155	76
No	49	24
Birth weight		
<2.5 kg	5	2.5
> = 2.5 kg	199	97.5
Gravidity		
Primigravidae	68	33.3
Gravid −2	48	23.5
Gravid 3 and above	88	43.1
Maternal age group		
Up to 20 yrs	52	25.5
Above 20 yrs	152	74.5
Education mother		
Up to primary	83	40.7
Post-primary	121	59.3

Of the study participants, 2.5% (n = 204) delivered low birth weight babies (< 2.5 kg), 5.9% delivered before 37 weeks of gestation while 91.6% delivered at term. Prevalence of *P. falciparum* parasitaemia (peripheral blood) among the mothers at delivery was 8.3% while 2.0% of the newborns had cord-blood parasitaemia. The majority (73.5%) of participants reported using iron sulphate and folic acid supplements during pregnancy.

The relationship between self-reported IPTp use and the general characteristics of the population are shown in Table 3. The more educated mothers (P = < 0.01 95% CI: 0.2-0.7) and those who took iron supplementation during their pregnancy (P = 0.03 95% CI:1.1-4.0) were more likely to report using IPTp. The other factors were not statistically different in the group that reported IPTp use and the cluster that reported IPTp non-use. None of the maternal demographic characteristics was associated with presence of sulphadoxine in mothers’ blood at delivery.

**Table 3:** Self-reported IPTp use during pregnancy and other demographic characteristics

**Variable**	**Used IPTp**	**Not used IPTp**	**P Value**	**(OR) 95%CI**
**Bed net use**				
Always	101	64		1
Sometimes	8	9	0.25	1.7(0.6-4.8)
Never	11	11	0.31	1.5(0.60-3.80)
**Education level**				
Up to primary	38	45		1
Post-primary	82	39	**<0.01**	0.4 (0.22-0.72
**Age group**				
Up to 20yrs	88	64	0.070	1.1 (0.6-2.2)
Above 20years	32	20		
**Iron supplement**				
Yes	98	57		1
no	22	27	**0.03**	2.1 (1.1-4.0)
**Use of insecticide spray**				
Yes	25	10		1
no	95	74	0.10	1.9 (0.88-4.3)
**Maternal parasitaemia**				
Negative	111	76		1
Positive	9	8	0.60	1.2 (0.47-3.51)

### Kappa statistic on self-reported IPTp use and sulphadoxine in blood at delivery

Of 120 study participants who self-reported to have used IPTp, 35 (29.2%) tested positive by HPLC while 63 (75%) of 84 patients who reported not to have used IPTp tested negative for SDX (see Table 1). On the other hand, 85 (70.8%) patients who reported to have used IPTp tested negative for blood SDX by HPLC. Yet, 21 (25%) of patients who self reported not to have taken SP were found to have detectable SDX in peripheral blood at delivery.

To determine agreement between self-report and HPLC detection of the drug in blood, Kappa analysis was used. The kappa statistic gives a numerical assessment of the degree at which two ratings or observers would actually agree compared to how much they would be in agreement just by chance. Assuming probability of observed agreement where P(a) is the percentage of agreement for self-reported IPTp use and SDX in blood, then by calculation, the probability of observed agreement, P(a) = (35 + 63)/204 = 0.48. The probability of expected agreement, P(e) is given by the formula: P(e) = [(n1/n) x (m1/n)] + [(n0/n) x (m0/n).

By substitution, P(e) = [{120/204}x{56/204}] + [{84/204}x{148/204}] =0.46.

Since Kappa, K = [p(a)-p(e)]/[1-p(e)], then by substitution, K = {0.48-0.46}/{1–0.46} = 0.037. This result (K = 0.037) signifies a very slight (poor-to-fair) agreement between reported IPTp use and SDX in blood at delivery [18,17].

## Discussion

This study explored the validity of self-reported sulfadoxine-pyrimethamine IPTp by testing for presence of SDX in maternal blood at delivery using HPLC. Two main findings of this study are that self-report on sulphadoxine-pyrimethamine IPTp use is unreliable not only for knowing whether the pregnant patient took the SP or not but also for finding out when the patient took the drug. Several patients who reported not having taken SP were found to have the drug derivatives in their blood. Further, some patients who reported having taken the drug before 9 weeks preceding baby delivery (when SDX would be too low to be detected in blood by HPLC) were also found to have the drug in the blood. On the other hand, some patients claiming to have taken SP within 9 weeks before delivery (when blood SDX would be detected by HPLC) actually did not have detectable SDX blood levels.

Interestingly, participants who self-reported IPTp use during their present pregnancy were more likely to have SDX in their circulation at delivery, although the level of agreement was only slight as assessed by kappa statistics. Although only 29.2% of participants who reported IPTp use actually had SDX in their blood at the time of delivery, 25% of participants who reported not taking IPTp had SDX in blood at delivery. This finding questions the validity of self-reported data in estimating the IPTp coverage. The findings of this study concur with a previous study in Uganda which found low validity of caretakers’ report on use of anti-malarials and antibiotics [11]. Okura *et al.* found that the young and more educated were more likely to report correctly on the prevalence of diseases such as hypertension, diabetes and history of myocardial infarction [19]. Another study found a good agreement of self report with diagnosis of diabetes and hypertension [13]. In another study in Kenya, which looked at anti-malarial drugs before initiating treatment in participants who reported no use of drug in 28 days prior to enrolment, it was found that the proportion of participants with residual anti-malarials was high and self-report on drug intake was unreliable [12]. Yet another study found that self reported compliance in use of antibiotics among sexually transmitted disease patients was also unreliable [20]. Thus, several previous studies concur with the findings of the present study where self-report data on IPTp use is only in slight agreement (kappa = 0.03) with results of HPLC detection of the drug ingredients in blood. In view of the demonstrable weakness of self reports, a previous study has suggested increasing the validity of self-reported data through focus group discussions, using language with which the respondents are very familiar, sequencing the questions from the least to the most threatening, using open-ended and direct questions [21]. In a review on validity of self reported data, Brener *et al.*[22] noted that validity can be improved when the patients understand the questions and are able to recall, their answers are anonymous and there is no fear of reprisals [22]. Using focus group discussions may reduce the fear of reprisals and increase anonymity, which improve validity of self-report. In contrast to household surveys, the present study was undertaken in a hospital setting, which may have led to selective recall bias for fear of possible repercussions. A previous review indicated that the validity of self-reported data may be affected by cognitive issues including clarity of the questions, memory needed to answer the questions and influence of the survey settings [22].

### IPTp use and other characteristics

Participants who reported IPT use during pregnancy tended to be younger in age, more educated, and reported having received iron supplementation during pregnancy (Table 3). In other words, the more educated participants were more likely to have reported IPTp intake than the less educated. There was a high ITN coverage (89.2%) in the participants, an encouraging finding which is important for prevention of malaria in pregnancy. This high bed net use could be due to the relatively high socio-economic status of the participants as the study population was largely urban and had access to contemporary distribution of ITNs to pregnant mothers free of charge. A recent study in Tanzania found that timely uptake of IPTp depends more on practices of health workers at the health units than individual characteristics of pregnant women and that early ANC attendance did not influence IPTp use [23]. Although 98.5% of the participants reported having attended the antenatal clinic at least once during that pregnancy, only 58.8% reported IPTp use during the current pregnancy.

This lower than expected use of IPTp could reflect suboptimal care at the antenatal clinics, lack of drugs in the health units and inadequate sensitization of the health workers which have been found to affect uptake [24]. Significantly, in areas with high transmission, pregnancy malaria still causes considerable morbidity and mortality in spite of high bed net use [25]. This emphasizes the importance of improving IPTp coverage to reduce the incidence and effects of pregnancy malaria efficiently.

Of the participants who reported non-use of IPT during pregnancy, 25% (n = 84) actually had the drug in circulation at the time of delivery. This finding suggests false reporting by the participants, which could be due to recall bias or the possibility that participants were not informed about the drugs given during the pregnancy, since the participants who reported using any sulpha drug during their pregnancy were excluded from the study.

## Conclusion

Current policy for control of pregnancy malaria emphasizes IPTp and use of insecticide-treated bed nets. To assess compliance with these recommendations and to estimate intervention coverage, RBM recommends the simpler and affordable approach of self-report in determining IPTp coverage. Blood drug levels are too costly for such population-based assessment. But, as shown by the present study, assessment of IPTp coverage by self-report is unreliable. Therefore, towards obtaining dependable data on IPTp coverage, the need to device ways of improving the accuracy of IPTp self-reports and the records that capture the data is very important.

### Limitations of the study

The study lacked precise information on the weight of the participants prevailing at the time of taking IPTp and relied on estimations basing on the weight of the mothers at delivery. Consequently, the C_max_ (SDX) for each patient could have been slightly overestimated for heavier mothers and *vice versa*. For a few participants (estimated at <5%), the time of taking the drug could not be accurately determined because of inexact recall of dates and incomplete case records. To the best of our knowledge, there are no previous similar studies of the validity of IPTp self-reports.

## Abbreviations

ANC, Antenatal Clinic; EDTA, Ethylene diamine tetra-acetic acid; HIV, Human Immune Deficiency Virus; HPLC, High Performance Liquid Chromatography; IPTp, Intermittent Presumptive Treatment; ITNs, Insectcide-treated Nets; RBM, Roll Back Malaria; SDX, Sulphadoxine; SP, Sulphadoxine + Pyrimethamine; UDHS, Uganda Demographic Health Survey; WHO, World Health Organization.

## Competing interests

The authors declare that they have no competing interests.

## Authors’ contributions

FN collected the clinical data, carried out data analysis and drafted the manuscript. MN carried out the high performance liquid chromatography and participated in design of the study. FM participated in the design of the study and performed the statistical analysis. MW and FK conceived of the study, participated in its design and coordination, and helped to draft the manuscript. All authors read and approved the final manuscript.
